# Influence of GDM Diagnosis and Treatment on Weight Gain, Dietary Intake and Physical Activity in Pregnant Women with Obesity: Secondary Analysis of the UPBEAT Study

**DOI:** 10.3390/nu12020359

**Published:** 2020-01-30

**Authors:** La’Shay Atakora, Lucilla Poston, Louise Hayes, Angela C. Flynn, Sara L. White

**Affiliations:** 1 London School of Hygiene and Tropical Medicine, London WC1E 7HT, UK; lashay.yeboa-atakora1@alumni.lshtm.ac.uk; 2 Department of Women and Children’s Health, King’s College London, London SE1 7EH, UK; lucilla.poston@kcl.ac.uk (L.P.); angela.flynn@kcl.ac.uk (A.C.F.); 3 Population Health Sciences Institute, Newcastle University, Newcastle upon Tyne NE2 4AX, UK; louise.hayes@newcastle.ac.uk; 4 Guy’s and St Thomas’ NHS Foundation Trust, London SE1 7EH, UK

**Keywords:** obesity, pregnancy, gestational diabetes, diet, physical activity, gestational weight gain

## Abstract

Obesity during pregnancy is associated with the development of gestational diabetes (GDM). This study aimed to assess if the result of an oral glucose tolerance test (OGTT) for GDM influences health (diet and physical activity) behaviours of pregnant women with obesity. In total, 1031 women who participated in the UK Pregnancies Better Eating and Activity Trial (UPBEAT) of a lifestyle intervention from early pregnancy were included. Changes in weight gain, dietary intake and physical activity following an OGTT undertaken between 27^+0^ and 28^+6^ weeks’ and 34 and 36 weeks’ gestation were examined using linear regression with appropriate adjustment for confounders. Obese women without GDM (IADPSG criteria) gained 1.9 kg (95% CI −2.2, −1.5, *p* < 0.001) more weight than women with GDM. Women with GDM demonstrated greater reductions in energy (–142kcal, 95%CI −242.2, −41.9, *p* = 0.006), carbohydrate intake (−1.5%E 95%CI –2.8, −0.3, *p* = 0.016) and glycaemic load (–15.2, 95%CI −23.6, –6.7, *p* < 0.001) and a greater increase in protein intake (2%E, 95%CI 1.3, 2.7, *p* < 0.001), compared to women without GDM. Trial intervention allocation did not influence any associations observed. The findings emphasise the need for strategies to optimise the health behaviours of pregnant women with obesity, following a negative OGTT for GDM.

## 1. Introduction

The recent increase in obesity in the UK population [1] is mirrored amongst women in antenatal care, with estimates suggesting that 23% have a BMI ≥ 30 kg/m^2^ [2]. Obesity in pregnancy increases the risk of complications [3], most notably, gestational diabetes (GDM), defined by new-onset hyperglycaemia in pregnancy, which now affects up to 30% of pregnancies worldwide [4]. Obesity in pregnancy is related to a 4–9-fold greater risk of GDM compared to pregnant women with a normal weight [5]. The long-term morbidities associated with GDM include progression to type 2 diabetes in approximately 25% of affected mothers [6].

The UK national guidelines for the management of GDM following diagnosis include the provision of advice on diet and physical activity. For women who do not gain adequate glycaemic control through changes in these behaviours, pharmacotherapy with metformin or insulin is prescribed [7]. 

Whilst differences in diet and weight gain between women with and without a diagnosis of GDM have been reported [8,9,10,11,12,13,14], there is a paucity of longitudinal data that explores behaviour change and gestational weight gain following a negative or positive oral glucose tolerance test (OGTT) for the diagnosis and associated treatment of GDM. In a recent longitudinal study of 702 women living in Norway, minimal differences in dietary intake between those with and without GDM were observed, and the women with GDM gained more weight compared to those without GDM [15]. To our knowledge, no previous study has investigated these associations in pregnant women with obesity. Understanding the behavioural patterns of obese women with and without a diagnosis of GDM following testing might help identify suboptimal health behaviours and inform future strategies to improve the health of pregnant women with obesity and their offspring.

The aim of this study was to assess how a negative or positive OGTT for the diagnosis and subsequent treatment of GDM influences the dietary intake, physical activity levels and gestational weight gain of pregnant women with obesity who were participants in UPBEAT, a randomised controlled trial of a lifestyle (diet and physical activity) intervention. We also determined if allocation to the intervention or control arm influenced the association between GDM status and behaviour change. We wished to explore, for example, whether an individual who was randomised to the active arm who subsequently did not develop GDM may show continued adherence to a lifestyle intervention.

## 2. Materials and Methods

### 2.1. Study Design and Population

This study is a secondary analysis of data collected from women who participated in the UK Pregnancies Better Eating and Activity Trial (UPBEAT). UPBEAT was a multicentre randomised controlled trial which took place in the UK. UPBEAT assessed whether a behavioural intervention of diet and physical activity advice reduced the incidence of GDM and the delivery of large-for-gestational-age (LGA) infants in pregnant women with obesity [16]. The protocol and main findings of UPBEAT have been previously published [16,17]. In brief, women were eligible to take part if they were aged 16 years or above, had a singleton pregnancy between 15^+0^ and 18^+6^ weeks’ gestation and had a body mass index (BMI) of 30 kg/m^2^ or above. Women were excluded if they did not give informed consent, were prescribed metformin, or if they had any pre-existing medical conditions. For the purposes of this investigation, only women who had an OGTT were included. Ethical approval was granted by the NHS Research Ethics Committee (UK Integrated Research Application System, reference 09/H0802/5). 

The UPBEAT intervention aimed to improve glucose tolerance through dietary and physical activity behaviour change. The participants randomised to the intervention group received eight weekly individual or group-based sessions, in addition to their standard antenatal care appointments. The dietary component of the intervention aimed to encourage a healthier eating pattern through a reduction in glycaemic load and saturated fat intake. To reduce glycaemic load, the participants were encouraged to swap high glycaemic index food and beverages for low glycaemic index alternatives and reduce the consumption of sugar-sweetened beverages including fruit juice. To reduce saturated fat intake, the participants were encouraged to use low fat dairy products and exchange fatty meats and meat products with leaner meat and fish. The physical activity component was tailored to participant preferences and focused on incremental increases in moderate intensity walking and being more active in daily life. Barriers to change were explored and specific, measurable, achievable, relevant, time-specific (SMART) goals were set for the women in the intervention arm. The participants randomised to the control arm were provided with antenatal care in line with local NHS guidelines [16]. We have previously reported that the intervention was not effective in reducing the prevalence of GDM or LGA infants, although several secondary outcomes including diet, weight gain and adiposity demonstrated evidence of improvement [17].

### 2.2. Data Collection 

The maternal social and demographic data obtained at enrolment included age (years), BMI (kg/m^2^), ethnicity (Black, White, Asian, other), parity (nulliparous, multiparous), smoking status (smoker, ex-smoker, non-smoker), living in a deprived area (Index of Multiple Deprivation (IMD); scores were calculated for the region of residence) and highest educational attainment. The data were obtained pre-intervention (15^+0^–18^+6^ weeks’ gestation), post-intervention (27^+0^–28^+6^ weeks’ gestation) and in late pregnancy (34–36 weeks’ gestation) for diet and physical activity (questionnaires), weight and anthropometric measures.

A semi-quantitative food frequency questionnaire (FFQ) adapted from one used in the UK arm of the European Prospective Investigation into Cancer Study (EPIC) was used to assess the diet of the participants for the preceding month. Glycaemic load was estimated based on the glycaemic index and carbohydrate content of each food, as reported previously [18]. Physical activity was assessed using the International Physical Activity Questionnaire (IPAQ) [16]. 

The trial protocol required OGTTs to be carried out at 27^+0^–28^+6^ weeks’ gestation. However, a more pragmatic approach was adopted to reflect clinical practice, and women who had OGTTs at 23–30 weeks’ gestation were included for the purposes of this analysis. GDM was diagnosed using International Association of Diabetes and Pregnancy Study Groups (IADPSG) criteria: fasting glucose of 5.1 mmol/L or higher, 1 h glucose of 10.0 mmol/L or higher, 2 h glucose of 8.5 mmol/L or higher, or a combination of these (venous blood; post 75 g glucose challenge) [19]. Women diagnosed with GDM were referred to the local antenatal diabetes service and managed according to local practice, with lifestyle dietary management, metformin or insulin treatment as appropriate.

### 2.3. Outcome Measures

Dietary outcomes included changes in total energy intake (kcal/day), glycaemic index, glycaemic load, carbohydrate (%E), protein (%E), total fat (%E), and saturated fat (%E) intake, from OGTT (27^+0^––28^+6^ weeks’ gestation) to late pregnancy (34–36 weeks’ gestation). Physical activity outcomes included change in moderate or vigorous activity (min/week) and walking (min/week), from OGTT (27^+0^–28^+6^ weeks’ gestation) to late pregnancy (34–36 weeks’ gestation). Anthropometric outcomes included change in weight (kg) from OGTT (27^+0^–28^+6^ weeks’ gestation) to late pregnancy (34–36 weeks’ gestation).

### 2.4. Statistical Analysis

The normality of the data was assessed using Shapiro–Wilk tests and visual representations including distributional diagnostic plots and histograms. Summary statistics were calculated for descriptive characteristics. Continuous variables were described as mean (standard deviation) or median (interquartile range). Categorical variables were described as number (percentage). Differences between characteristics at study entry were examined using chi-squared tests for categorical data, and independent sample t-tests for continuous data. The proportion of women who gained weight above or below the National Academy of Medicine (NAM) guidelines for pregnant women with obesity was determined using NAM guidance for weekly weight gain in the third trimester [20]. Univariate and multivariable linear regression were used to examine the association between GDM diagnosis and the change in each outcome variable between 27^+0^ and 28^+6^ weeks’ gestation and 34 and 36 weeks’ gestation. Models were adjusted for maternal BMI, ethnicity and neonatal sex. To identify whether intervention allocation was an effect modifier on the association between GDM status and each outcome, an interaction term was added to the linear regression analyses. Likelihood ratio tests were conducted to ascertain the statistical significance of the interaction.

Statistical analyses were performed using Stata version 16.0 (StataCorp LP, College Station, TX, USA).

## 3. Results

**Figure 1:**
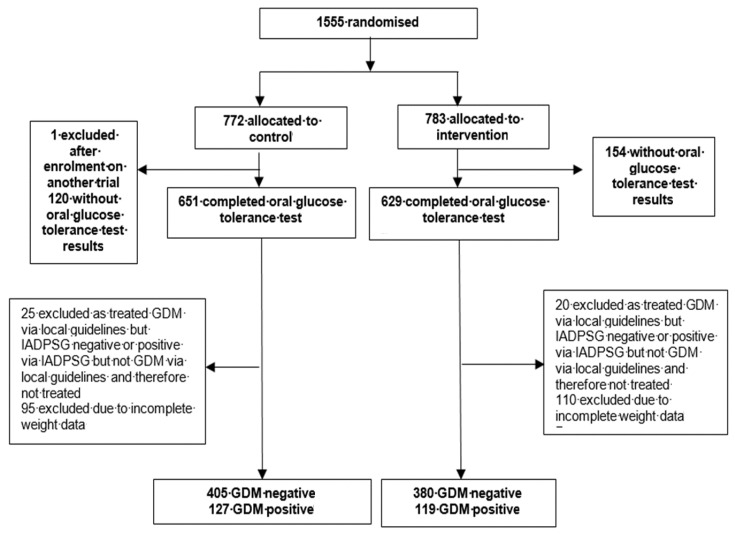
Study profile.

Between March 2009 and June 2014, 1555 women were randomised to either the behavioural intervention or standard antenatal care. The present investigation was limited to those participants who received OGTTs (*n* = 1031 participants, 66.3%) (Figure 1).

### 3.1. Study Population

The characteristics of the study population are shown in Table 1. Two hundred and forty-six participants (23.9%) were diagnosed with GDM by OGTT. The non-GDM women were younger at study entry, weighed less, and had a lower BMI than the GDM women (all *p* < 0.05). GDM women were more likely to have a history of GDM. The GDM and non-GDM women were similar in smoking status, ethnicity, parity, education level and IMD quintile. Infants born to GDM women were more likely to be large-for-gestational-age (*p* < 0.05).

### 3.2. Gestational Weight Gain 

**Figure 2:**
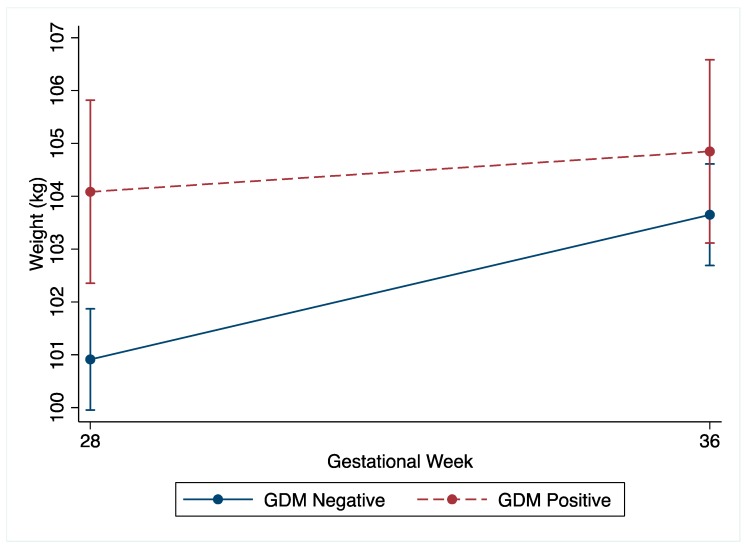
Unadjusted weight (kg) change of GDM and non-GDM women from 27^+0^–28^+6^ weeks’ gestation (28 weeks) to 34–36 weeks’ gestation (36 weeks) with 95% confidence intervals.

The non-GDM women demonstrated greater total gestational weight gain compared to the GDM women (non-GDM: mean 8.0 kg (SD 4.3) vs. GDM: 5.8 kg (SD 4.5), *p* < 0.001). Following the OGTT at 27^+0^–28^+6^ weeks’ gestation, the change in weight by 34–36 weeks’ gestation was greater in the non-GDM women (Table 2 and Figure 2). The non-GDM women gained 1.9 kg (95%CI −2.2, −1.5, *p* < 0.001) more than the GDM women, which was robust to adjustment for confounders (Supplementary Figure S1A). Amongst the non-GDM women, 60.3% (*n* = 473) gained weight above NAM recommendations for third trimester weight gain compared to 26.4% (*n* = 65) of the GDM women. Non-GDM women were also less likely to gain inadequate weight, with 0.6% (*n* = 149) gaining less weight than NAM recommendations, compared to 26.6% (*n* = 206) amongst the GDM women. Although underpowered, no differences in LGA or SGA were noted between the non-GDM women who gained within the NAM guidelines and those who gained above the NAM guidelines (Supplementary Tables S1 and S2).

### 3.3. Dietary Intake

**Figure 3:**
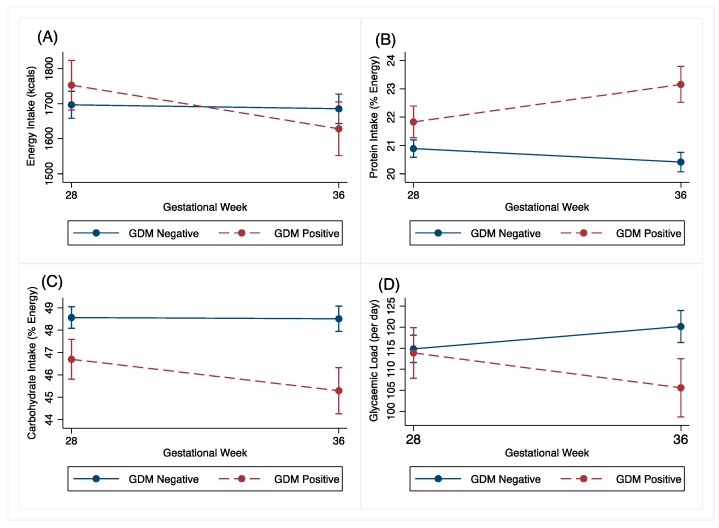
Unadjusted (**A**) energy (kcals), (**B**) protein (% energy), (**C**) carbohydrate (% energy) and (**D**) glycaemic load change of GDM and non-GDM women from 27^+0^–28^+6^ weeks’ gestation (28 weeks) to 34–36 weeks’ gestation (36 weeks), with 95% confidence intervals.

Changes in dietary intake between the GDM and non-GDM women following the OGTT at 27^+0^–28^+6^ weeks’ gestation to 34–36 weeks’ gestation are shown in Table 2 and Figure 3. The GDM women reduced their energy intake by 142kcal (95% CI −242.2, −41.9, *p* = 0.006) more than the non-GDM women. The GDM women also reduced their carbohydrate intake by 1.5%E (95% CI −2.8, 0.3, *p* = 0.016) and their glycaemic load by 15.2%E (95% CI −23.6, −6.7, *P* < 0.001) more than the non-GDM women. The GDM women changed their protein intake by 2%E (95% CI 1.3, 2.7, *p* < 0.001) more than the non-GDM women in the same time period. These associations were robust to adjustment for confounders. Adjusted graphs are presented in Supplementary Figures S1B–E.

There were no differences in change in total fat intake, saturated fat intake or glycaemic index between the GDM and non-GDM women (Table 2). 

### 3.4. Physical Activity Changes

There were no differences in change in vigorous and moderate activity and walking between the GDM and non-GDM women (Table 2).

### 3.5. Intervention Allocation

Allocation to either the intervention or control group did not modify the association between GDM status and all outcomes.

## 4. Discussion

This study found that a higher proportion of pregnant women with obesity who were not diagnosed with GDM gained weight in excess of recommended gestational weight gain in their last trimester of pregnancy. In contrast, the diagnosis and treatment of GDM were associated with lower weight gain and dietary change.

Consistent with our findings, previous studies in women with heterogeneous BMIs assessing weight gain in the interval after GDM screening have found that women without GDM gain more weight than women with GDM [12,14]. In a small US study of 89 women, Chakkalakal et al. reported that women with GDM gained weight at a lower rate than women without GDM (0.30 ± 0.28 kg/week vs. 0.53 ± 0.28 kg/week, *p* = 0.001) [12]. Similarly, women with GDM gained less total weight than women without GDM in a study of 212 Australian women, when weight was assessed in the second and third trimester (GDM: 1.18 kg (1.6%) vs. non-GDM: 4.0 kg (4.8%), *p* < 0.001) [14].

As additionally seen in this study, it is well known that women diagnosed with GDM are more likely to give birth to LGA infants despite dietary changes, physical activity and pharmacological approaches, supporting earlier intervention in such women. Despite a higher prevalence of LGA infants in the GDM group, the finding that a high proportion of obese women without GDM gained weight in excess of the NAM guidelines in the third trimester is of particular concern in a population that already has an increased risk of complications [3]. In the German Programming of Enhanced Adiposity Risk in Childhood–Early Screening (PEACHES) study, excessive third trimester weight gain in women without GDM was related to late-pregnancy dysglycaemia [21]. The evidence from this study suggests that pregnant women with obesity may benefit from weight management advice following OGTT, regardless of GDM diagnostic classification.

In the current study, 26% of women with GDM gained weight which was below the NAM recommendations. Total gestational weight gain below NAM recommendations has been associated with a reduced risk of peripartum complications for pregnant women with obesity but an increased risk of complications in their neonates, including low birth weight, preterm delivery and neonatal mortality [22]. Further research is required to investigate the association between lower third trimester weight gain and pregnancy outcomes in women with GDM who are obese during pregnancy.

Internationally, guidelines for the management of GDM recommend that women be offered dietary advice to improve glycaemic control. This is the most likely explanation for the greater improvement in dietary intake in the women diagnosed with GDM in this study compared to the women without GDM. This contrasts to that reported by Elvebakk et al. in Norwegian women, who showed only marginal differences in dietary intake between women with and without GDM from 18–22 weeks’ gestation to 32–36 weeks’ gestation [15]. Two cohort studies, in the UK and the US, have reported that women with GDM had lower energy intakes than women without GDM [8,11], similar to the present study. Others have reported that women with GDM had lower intakes of total and saturated fats compared with women without GDM [8,10]. The absence of any changes in total or saturated fat between the groups in the present study may be explained by the focus in the UK on carbohydrate intake to optimise blood glucose control [7]. The difference in weight gain is also likely explained by the management of women with GDM following diagnosis which includes close monitoring of gestational weight gain as well as promotion of dietary change [7,23]. Women with GDM additionally receive regular feedback through the monitoring of blood glucose concentration which can inform dietary choices. Furthermore, they are potentially aware of the risks, particularly to their baby, associated with GDM, which may motivate behaviour change. Women who are not diagnosed with GDM do not have the same motivators; indeed, poor food choices may be positively enforced by apparent lack of disease [24].

There is a paucity of evidence on the association between GDM status and physical activity, with conflicting reports [25,26]. This study identified no differences in physical activity levels between women with and without GDM in the interval following GDM screening. The lack of change in physical activity for both groups aligns with reported barriers to physical activity for such women including a lack of time, pain, fatigue, work and childcare commitments [27].

Whilst it might be hypothesised that randomisation to the intervention arm followed by a diagnosis of GDM might influence the attitude to behavioural change, we found that allocation to a behavioural intervention in early pregnancy did not influence the association between GDM diagnosis and an associated change in diet and physical activity.

This study has several strengths. It is the first to examine the association between GDM status and health behaviours in a cohort of obese pregnant women. The detailed UPBEAT study database provided the opportunity to explore relationships in the interval following GDM screening to late gestation, whereas previous investigations have depended predominantly on cross-sectional data.

Limitations include the collection of dietary intake and physical activity data by self-report questionnaires which may be prone to recall bias [28]. The original trial was not powered to investigate the association between changes in health behaviours and weight gain and pregnancy outcomes. Data were collected in women taking part in a clinical trial, which may have introduced selection bias.

## 5. Conclusions

Whilst this study confirms that the current guidelines to treat women with GDM are effective in promoting behaviour change and limiting weight gain in obese women diagnosed and treated for GDM, we highlight the unintended consequence of a suboptimal diet and greater weight gain associated with not being diagnosed with GDM. We emphasise the need for strategies to manage dietary intake and gestational weight gain in pregnant women with obesity who receive a negative OGTT result, in order to reduce the risk of adverse health outcomes for mother and child.

## Figures and Tables

**Table 1:** Characteristics at 15^+0^–18^+6^ weeks’ gestation of the study population who had an oral glucose tolerance test.

	Whole Group *n* = 1031 (%)	Non-GDM *n* = 785 (76.1%)	GDM ^a^ *n* = 246 (23.9%)	*p* ^d^
**Age (years)**	30.9 (5.4)	30.4 (5.5)	32.3 (4.9)	<0.001
**Ethnicity**				
White	688/1031 (66.7)	532/785 (67.8)	156/246 (63.4)	0.628
Black	231/1031 (22.4)	169/785 (21.5)	62/246 (25.2)	
Asian	56/1031 (5.4)	42/785 (5.4)	14/246 (5.7)	
Other	56/1031 (5.4)	42/785 (5.4)	14/246 (5.7)	
**Weight (kg)**	98.0 (15.0)	97.4 (14.2)	99.9 (17.2)	0.024
**BMI (kg/m^2^)**	35.4 (4.8)	35.2 (4.6)	36.0 (5.3)	0.025
**Education** ^b^				0.175
None or GCSE	202/1031 (19.6)	150/785 (19.1)	52/246 (21.1)	
Vocational qualification	243/1031 (23.6)	175/785 (22.3)	68/246 (27.6)	
A-level (or equivalent)	164/1031 (15.9)	132/785 (16.8)	32/246 (13.0)	
First or higher degree	422/1031 (40.9)	328/785 (41.8)	94/246 (38.2)	
**Index of multiple deprivation** ^c^				0.118
1 (least deprived)	26/820 (3.2)	20/618 (3.2)	6/202 (3.0)	
2	40/820 (4.9)	32/618 (5.2)	8/202 (4.0)	
3	100/820 (12.2)	81/618 (13.1)	19/202 (9.4)	
4	309/820 (37.7)	241/618 (39.0)	68/202 (33.7)	
5 (most deprived)	345/820 (42.1)	244/618 (39.5)	101/202 (50.0)	
**Current smoker**	62/1031 (6.0)	42/785 (5.4)	20/246 (8.1)	0.460
**Multiparous**	560/1031 (54.3)	417/785 (53.1)	143/246 (58.1)	0.169
**Previous history of GDM**	17/560 (3.0)	6/417 (1.4)	11/143 (7.7)	<0.001
**Randomised group**				0.993
Intervention	499/1031(48.4)	380/785 (48.4)	119/246 (48.4)	
Standard care	532/1031 (51.6)	405/785 (51.6)	127/246 (51.6)	
				
**Neonatal characteristics**				
**Neonatal sex**				
Male	534/1031 (51.8)	410/785 (52.2)	124/246 (50.4)	0.618
Gestational age at delivery (weeks)	39.8 (1.4)	40.1 (1.3)	38.9 (1.2)	<0.001
Birthweight (g)	3482 (493)	3509 (499)	3397 (463)	0.002
**Customised birthweight centiles** ^e^				
≥90th (LGA)	85/1031 (8.2)	55/785 (7.0)	30/246 (12.2)	0.010
≤10th (SGA)	117/1031 (11.4)	95/785 (12.1)	22/246 (8.9)	0.173

Values are mean (standard deviation) or number (%); a GDM, Gestational Diabetes Mellitus; b GCSE, General Certificate of Secondary Education, A-level, General Certificate of Education Advanced level; c Index of multiple deprivation is a measure of relative deprivation. Scores calculated for region of residence by fifths of the population. UK-wide scores were developed from English and Scottish data relating to employment and income domains [17]; d P value comparing the difference between GDM and non-GDM women. Obtained through independent sample t-test for continuous variables and chi-squared for categorical variables; e Large-for-gestational-age (LGA) as >90th customised birthweight centile for gestational age, adjusted for maternal height and weight, ethnic origin, parity, and sex of the baby, and small-for-gestational-age (SGA) as ≤10th customised birthweight centile.

**Table 2:** Weight gain, dietary intake and physical activity outcomes for the GDM and non-GDM women by gestational period, and unadjusted and adjusted linear regression models examining the association between GDM diagnosis and change in each outcome variable between 27+0–28+6 weeks’ and 34–36 weeks’ gestation.

Outcome	Non-GDM	GDM	Unadjusted Coefficient (95% CI)	*p*	Adjusted Coefficient (95% CI)	*p*
**Weight gain**						
Weight (kg)						
27^+0^–28^+6^ weeks	101.4 (14.1)	103.6 (17.5)				
34–36 weeks	104.1 (14.3)	104.4 (18.0)	–2.0 (–2.3, –1.6)	<0.001	–1.9 (–2.2, –1.5)	<0.001
**Dietary intake**						
Energy intake (kcal)						
27^+0^–28^+6^ weeks	1729.9 (527.6)	1809.8 (507.6)				
34–36 weeks	1721.8 (528.4)	1658.8 (499.2)	–142.9 (–241.7, –44.1)	0.005	–142.0 (–242.2, –41.9)	0.006
Total fat intake (%E)						
27^+0^–28^+6^ weeks	31.0 (5.1)	31.9 (5.0)				
34–36 weeks	31.2 (5.0)	31.6 (5.2)	–0.5 (–1.4, 0.4)	0.253	–0.5 (–1.4, 0.4)	0.318
Saturated fat intake (%E)						
27^+0^–28^+6^ weeks	12.7 (2.9)	13.0 (2.9)				
34–36 weeks	12.9 (2.8)	12.8 (2.7)	–0.4 (–0.9, 0.1)	0.082	–0.4 (–0.9, 0.1)	0.086
Protein intake (%E)						
27^+0^–28^+6^ weeks	21.0 (4.3)	22.0 (4.4)				
34–36 weeks	20.5 (4.3)	23.5 (4.8)	1.9 (1.3, 2.6)	<0.001	2.0 (1.3, 2.7)	<0.001
Carbohydrate intake (%E)						
27^+0^–28^+6^ weeks	48.1 (6.2)	46.1 (6.8)				
34–36 weeks	48.4 (6.5)	45.0 (7.5)	–1.4 (–2.7, –0.2,)	0.021	–1.5 (–2.8, –0.3)	0.016
Glycaemic index						
27^+0^–28^+6^ weeks	55.8 (4.0)	55.1 (4.2)				
34–36 weeks	56.1 (4.1)	55.0 (4.5)	–0.3 (–1.0, 0.3)	0.279	–0.4 (–1.0, 0.3)	0.272
Glycaemic load						
27^+0^–28^+6^ weeks	124.7 (46.3)	123.1 (43.1)				
34–36 weeks	125.5 (46.1)	108.8 (36.0)	–15.1 (–23.4, –6.7)	<0.001	–15.2 (–23.6, –6.7)	<0.001
**Physical activity**						
Moderate or vigorous activity (min/week)						
27^+0^–28^+6^ weeks	30 (0–240)	15 (0–240)				
34–36 weeks	0 (0–180)	0 (0–180)	–0.02 (–0.4, 0.4)	0.915	–0. 00008 (–0.4, 0.4)	1.0
Walking (min/week)						
27^+0^–28^+6^ weeks	360 (150–630)	300 (120–630)				
34–36 weeks	300 (140–600)	280 (120–560)	–0.2 (–0.5, 0.1)	0.244	–0.2 (–0.5, 0.1)	0.162

Data are presented as mean (SD) or median (IQR). The weight gain, dietary intake and physical activity estimates (coefficients) were calculated using linear regression and adjusted for BMI at study entry, ethnicity and neonatal sex. The weight gain analyses included 1031 women, of whom 785 did not have GDM and 246 had GDM. There were 653 women included in the dietary intake analysis; 507 did not have GDM and 146 had GDM. The physical activity outcomes were log transformed and the estimates were calculated using linear regression. For the physical activity analyses, 236 women were included in the unadjusted and adjusted analyses; 181 women did not have GDM and 55 had GDM.

## Supplementary Materials

The following are available online at https://www.mdpi.com/2072-6643/12/2/359/s1, Figure S1: (**A**–**E**), Adjusted weight (kg) change of GDM and non-GDM women from 27^+0^–28^+6^ weeks’ gestation (28 weeks) to 34–36 weeks’ gestation (36 weeks), with 95% confidence intervals. Adjusted for maternal BMI, ethnicity and neonatal sex; Table S1: Frequency of neonatal birthweight complications for non-GDM women by NAM gestational weight gain guidelines, Table S2: Neonatal birthweight complications for non-GDM women by NAM gestational weight gain guidelines.
