# Supplementary material for: Influence of GDM Diagnosis and Treatment on Weight Gain, Dietary Intake and Physical Activity in Pregnant Women with Obesity: Secondary Analysis of the UPBEAT Study
Source: Nutrients. 2020 Jan 30;12(2):359. doi: 10.3390/nu12020359 (PMC7071182; doi:10.3390/nu12020359)
Supplement: Supplementary file 1 [file nutrients-12-00359-s001.pdf]

Supplementary Table S1: Frequency of neonatal birthweight complications for non-GDM women by NAM gestational weight gain guidelines.

	N=785	Gestational weight gain below NAM guidelines	Gestational weight gain within NAM guidelines	Gestational weight gain above NAM guidelines
**LGA (>90th)**	55/785	14 (25.4)	4 (7.3)	37 (67.3)
**LGA (>95th)**	23/785	6 (26.1)	2 (8.7)	15 (65.2)
**SGA (<10th)**	95/785	34 (35.8)	8 (8.4)	53 (55.8)
**SGA (<5th)**	51/785	22 (43.1)	2 (3.9)	27 (53.0)

Gestational weight gain between 27^{+0}-28^{+6} weeks' and 34-36 weeks' gestation. NAM, National Academy of Medicine

Supplementary Table S2: Neonatal birthweight complications for non-GDM women by NAM gestational weight gain guidelines.

	Gestational weight gain within NAM guidelines	Gestational weight gain above NAM guidelines	*P*
**LGA (>90th)**	4/106 (3.8)	37/473 (7.8)	0.142
**LGA (>95th)**	2/106 (1.9)	15/473 (3.2)	0.479
**SGA (<10th)**	8/106 (7.6)	53/473 (11.2)	0.268
**SGA (<5th)**	2/106 (1.9)	27/473 (5.7)	0.103

Gestational weight gain between 27^{+0}-28^{+6} weeks' and 34-36 weeks' gestation. *P* value obtained using chi-squared test. NAM, National Academy of Medicine

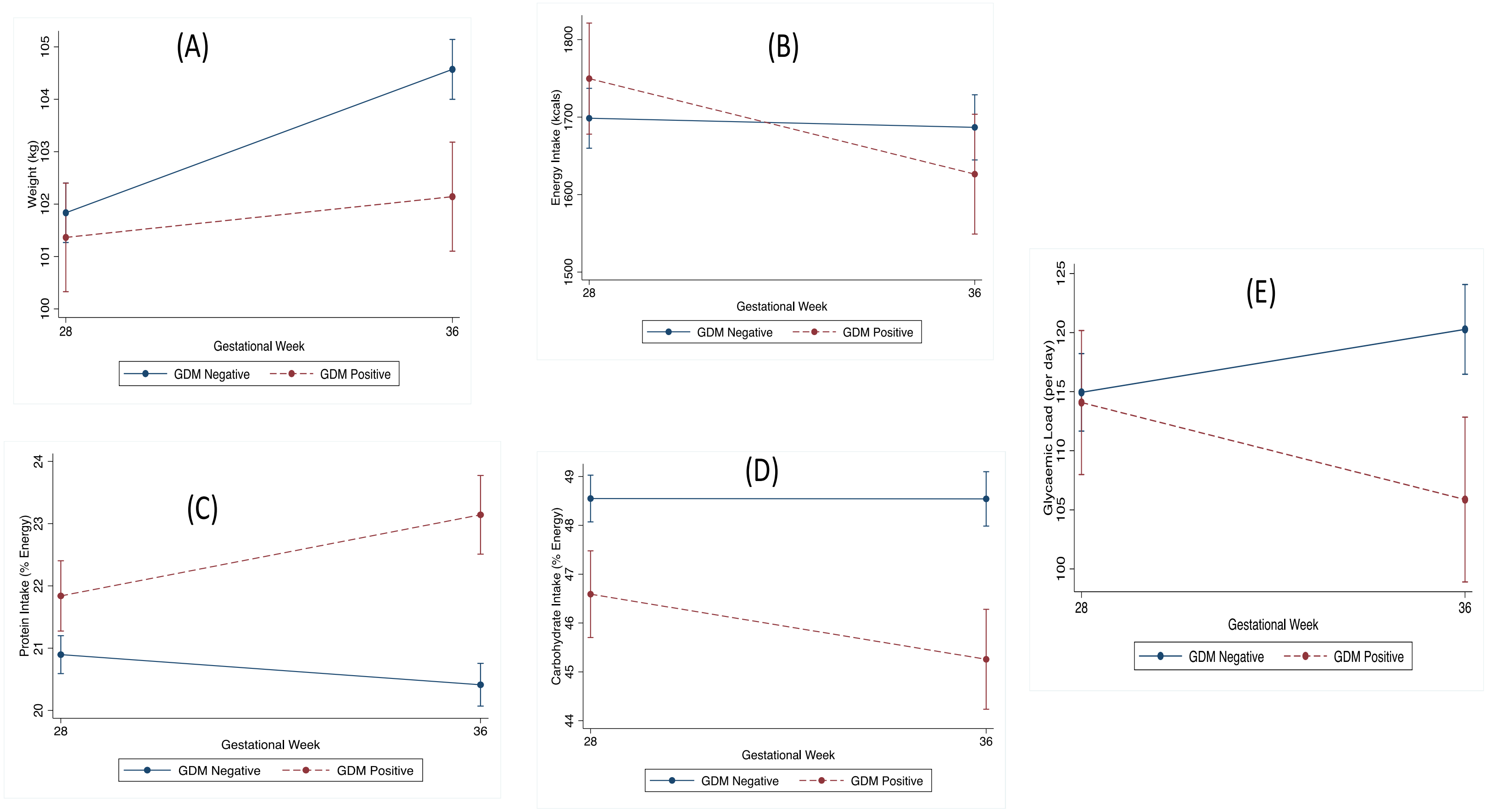

Supplementary Figure S1. (A-E). Adjusted weight (kg) change of GDM and non-GDM women from 27^{+0}-28^{+6} weeks' gestation (28 weeks) to 34-36 weeks' gestation (36 weeks), with 95% confidence intervals. Adjusted for maternal BMI, ethnicity and neonatal sex.
